# Evaluating acute stress responses to height: validity of heart rate variability, respiratory markers, and competitive state anxiety inventory

**DOI:** 10.3389/fpsyg.2025.1597839

**Published:** 2025-08-19

**Authors:** Lukáš Psohlavec, Mike Rogerson, Tomáš Brtník, Jiří Baláš

**Affiliations:** ^1^Faculty of Physical Education and Sport, Charles University, Prague, Czechia; ^2^School of Sport, Rehabilitation and Exercise Sciences, University of Essex, Colchester, United Kingdom

**Keywords:** ropes course, CSAI-2R oxygen consumption, respiration, metabolic cost, height

## Abstract

Despite the widespread use of height exposure in adventure-based programs to foster resilience, effectiveness has largely been evaluated through self-reported measures, with limited objective psychophysiological assessments. This study aimed to identify which physiological stress markers best determine the acute stress response to height. A secondary aim of the study was to assess the concurrent validity of the Competitive State Anxiety Inventory-2 Revised (CSAI-2R) questionnaire with ventilatory and heart rate variability (HRV) markers. A total of 55 healthy university students participated in a controlled experiment involving three walks on a log positioned at varying heights (0.3 m and 10.5 m). Psychometric measures were recorded using the Competitive State Anxiety Inventory-2 Revised, while physiological responses were monitored through HRV and respiratory markers. High-obstacle conditions significantly increased somatic (↑6.1 ± 5.7; *p* < 0.01) and cognitive anxiety (↑2.9 ± 5.9; *p* < 0.01) while reducing self-confidence (↓3.0 ± 5.6; *p* < 0.01). Based on the effect size (*η*_p_^2^), the largest differences between low- and high-height conditions for physiological markers were observed in heart rate (HR) (*η*_p_^2^ = 0.910), ventilation (*η*_p_^2^ = 0.906), oxygen uptake (*η*_p_^2^ = 0.891; *p* < 0.001), and tidal volume (VT) (*η*_p_^2^ = 0.872). Smaller differences were found for HRV markers, including the parasympathetic nervous system (PNS) index (*η*_p_^2^ = 0.860) and the sympathetic nervous system (SNS) index (*η*_p_^2^ = 0.798). Notably, weak correlations were observed between physiological markers and self-reported anxiety measures (*R* = −0.454 to 0.323), raising questions about the concurrent validity of psychometric tools. The findings suggest that while height exposure induces a pronounced stress response, the combination of HR and respiratory measures with psychological tools provides a more comprehensive understanding of stress coping during height exposure.

## Introduction

Based on the doxa assumption that height is stressful for many individuals, exposure to height via adventure activities—such as high ropes courses, climbing, and abseiling—is often used in outdoor adventure-based programs as a challenging and potentially stressful experience for educational purposes. Acute stress-related physiological and psychological responses are often measured, as they can provide valuable insights into stress coping mechanisms and resilience (Bailey et al., 2017; Baláš et al., 2017; Bunting et al., 2000; Bunting and Gibbons, 2001; Davidson et al., 2016; Draper et al., 2010; Ewert et al., 2016; Gajdošík et al., 2020).

### Physiological markers of stress

More broadly, acute physiological responses to adventure activities have been assessed using salivary cortisol (Draper et al., 2010; Ewert et al., 2016; Magiera et al., 2018), urine catecholamines (Bunting et al., 2000), EEG activity (Bailey et al., 2017), venous blood catecholamines and cortisol (Baláš et al., 2017; Bunting and Gibbons, 2001), heart rate (HR) (Draper et al., 2010; Giles et al., 2020; Magiera et al., 2018; Watts et al., 1999), and respiratory markers (Draper et al., 2010; Gajdošík et al., 2020; Giles et al., 2020; Watts et al., 1999).

While hormones from both the hypothalamic–pituitary–adrenal (HPA) axis and the sympathetic-adrenal-medullary axis reflect the stress response, the use of cortisol can be challenging due to individual variation in reactivity (i.e., how long it takes for an associated peak in cortisol to be reached after a stressor), especially when data are collected at a set time following the event of interest, individual variation in diurnal rhythms, and the expense of processing samples. The use of other catecholamines can be problematic in outdoor settings due to rapid degradation after stress cessation (Ewert, 2015; Zouhal et al., 2008).

Therefore, utilizing heart rate, respiratory markers, and heart rate variability (HRV) is often a more financially and logistically viable way for assessing the stress response in outdoor settings such as adventure activities. For instance, HRV has been shown to reflect vagal activity in these settings (Gladwell et al., 2016), and heart rate is a frequently used measure in sports and exercise sciences and has also been shown to respond to various stressors (Taelman et al., 2009).

Therefore, the current study sought to address the literature gap regarding physiological markers of stress measured during an adventure activity at elevated heights.

### Psychological markers of stress and their validity

Either to focus on the mental aspect or to triangulate and provide a more holistic understanding of the potentially stressful activity, psychological self-report is often employed. Anxiety responses, which are of particular interest in adventure activity-based programs, can be assessed using a range of questionnaires, such as the Competitive State Anxiety Inventory-2 (CSAI-2) (Baláš et al., 2017; Draper et al., 2010; Giles et al., 2020), State–Trait Anxiety Inventory (STAI), Anxiety Thermometer (Pijpers et al., 2003, 2005), and Spielberg State Anxiety Inventory (SSAI) (Bunting and Gibbons, 2001). Anxiety is an emotion characterized by feelings of tension, worried thoughts, and physical changes in the body (Foa et al., 2005). It is made up of multiple components, although the exact number and nature of these components remain a topic of debate in the literature (Brodbeck et al., 2011; Clark and Watson, 1991). The CSAI-2 questionnaire models anxiety as comprising three components—cognitive, somatic, and self-confidence. Although stress and anxiety are interrelated, it makes sense that for adventure activities at height, anxiety—as a future-oriented state—will likely be most pertinent before performing the activity, whereas stress will be more pertinent during the performance.

Although combinations of physiological markers and self-reported anxiety are frequently used in research and practice, very little is currently known about the concordant validity between psychometric tools such as the CSAI and some physiological markers of stress (Goyal et al., 2016), particularly when they are being used in adventure domains. In activity-based adventure programs, participants often report or are observed to experience high anxiety immediately before the activity at height. It would be useful for practitioners to understand the extent to which pre-activity anxiety translates into stress during the activity itself. Therefore, the secondary aim of this study was to assess the concordant validity of the Competitive State Anxiety Inventory-2 Revised (CSAI-2R) questionnaire with respiratory and cardio markers of stress in order to understand the extent to which stress and anxiety are separate ‘constructs’ and whether self-reporting can be used as a proxy measure for physiological markers.

## Materials and methods

### Participants

A total of 55 university students (weight 72.2 ± 11.2 kg, height 177.1 ± 8.1 cm, age 20.3 ± 2.1 years) participated voluntarily in this study. They were healthy and asked to refrain from high-intensity exercise (24 h), caffeine (12 h), and heavy meals (2 h) prior to the experiment. All participants signed an informed consent at the beginning of the study. The local university’s ethics committee granted approval for the study (EK238/2020). All participants participated voluntarily.

### Study design

All participants completed three 4-min walks on a narrow log at two different heights. The first walk took place on a log 0.3 m above the ground at a self-selected speed and was used only for psychometric assessments. The second walk was conducted at 10.5 m above the ground, also at a self-selected speed. The third walk was performed again at 0.3 m above the ground, but this time at the same speed as the high-obstacle trial, ensuring a consistent walking speed for subsequent physiological comparisons.

Randomization of the conditions was not possible for the following reasons:

- The first walk at the low-obstacle height served as a familiarization trial with the log, aiming to reduce stress caused by a lack of skills needed to overcome the obstacle.
- Standardizing walking speed across conditions was essential for accurately assessing physiological responses, as walking speed is a key factor influencing physiological changes. Since it was not possible to control the walking speed during the high-obstacle trial due to participants’ heightened stress responses, the high-obstacle trial had to be completed first. This allowed the walking speed from the high-obstacle trial to be replicated during the second low-obstacle trial, ensuring consistency for physiological comparisons.
- Administering the CSAI-2R questionnaire during the low-obstacle condition after the high-obstacle trial could have introduced bias, as the participants’ successful completion of the high-obstacle walk may have influenced their responses.

**Figure 1:**
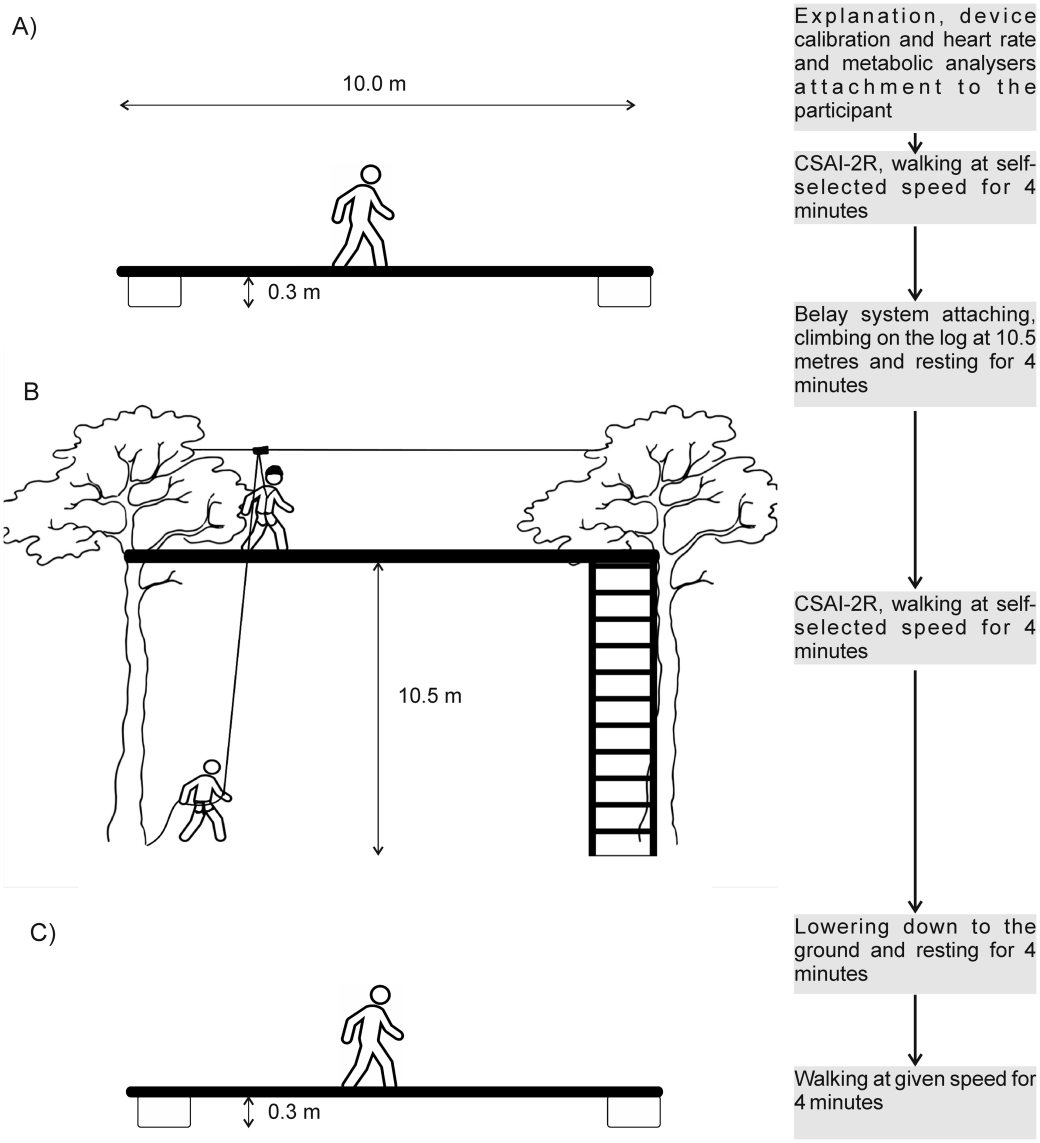
Design of the study. **(A)** Walking at a self-selected speed on a log 0.3 m above the ground. **(B)** Walking at a self-selected speed on a log 10.5 m above the ground. **(C)** Walking at a given speed on a log 0.3 m above the ground.

A portable metabolic system was worn on the chest, and anxiety metrics were recorded during all three conditions, as depicted in Figure 1. Participants completed the CSAI-2R immediately before the first and the second walk.

### High- and low-obstacle courses

The same two rounded wooden logs (13 m long, 0.25 m in diameter) were placed at 0.3 m (low-obstacle) and 10.5 m (high-obstacle) above the ground, respectively. The middle 10-m section was marked at 1-m intervals using colored marks. The participants wore a full-body harness during both low- and high-obstacle conditions. However, only during the high-obstacle condition, the participants were belayed by an experienced instructor using a preinstalled rope. The rope was fixed on the participants’ backs so that they could not see it. The rope passed through a carabiner with a pulley, which was running freely on a metallic wire 4 m above the log. From the carabiner, the rope went back to the belayer at the ground level. To control the walking speed during the third walk, the bottom log was highlighted with colored marks every meter, and a researcher verbally guided the participants to accelerate or decelerate their walking pace. At the end of the log, a small balance support attached to a tree was provided to assist with turning.

### Physiological responses

Physiological responses were assessed using an indirect calorimetry open system and HRV.

#### Gas analysis

A portable breath-by-breath metabolic system (MetaMax 3B, Cortex Biophysik, Germany) was worn by the participants using a chest harness (total weight 1.4 kg). Gas calibration was performed with a reference gas (15% O_2_ and 5% CO_2_), and volume calibration was performed using a 3-L syringe. Ambient air calibration was performed before each measurement. Breath-by-breath data were averaged over 20-s intervals for oxygen uptake (VO_2_), carbon dioxide production (VCO_2_), expiratory ventilation (V_E_), and breath frequency (BF), then exported to Excel for further analysis.

#### Heart rate variability

A chest belt (Polar Electro H10 OY, Finland) was used for monitoring heart rate (HR) and HRV. Data were measured continuously and stored using a Polar watch (Polar Grit X Pro, Kempele, Finland), then they were exported and analyzed using the Kubios HRV software (Biosignal Analysis and Medical Imaging Group, Kuopio, Finland). The analyzed HRV markers were selected according to previous recommendations for short-term HRV analysis (Castaldo et al., 2015; Kim et al., 2018; Pereira et al., 2017; Shaffer and Ginsberg, 2017) and were as follows.

#### Time-domain parameters

Mean heart rate (HR), average time between consecutive heartbeats (RR), root mean square of successive differences between RR intervals (RMSSD), number of successive RR interval pairs that differ by more than 50 ms (NN50), stress index (SI), and mean of the standard deviations of RR intervals in 5-min segments (SDNN).

#### Frequency-domain parameters

Absolute low-frequency power (0.04–0.15 Hz) measured using the fast Fourier transform (FFT) (LFpow_FFT (ms^2^)), high-frequency power (0.15–0.4 Hz) measured using the FFT (HFpow_FFT (ms^2^)), normalized low-frequency power expressed in normalized units (LFpow_FFT (n.u.)), high-frequency power expressed in normalized units (HFpow_FFT (n.u.)), and low-frequency power to high-frequency power ratio (LF/HF Ratio (FFT)).

#### Non-linear HRV parameters

Standard Deviation 1 (SD1): a measure of short-term HRV, specifically derived from the Poincaré plot, and detrended fluctuation analysis, Alpha 1: a non-linear HRV measure that assesses short-term correlations in the time series of heart rate data (DFA1).

#### Parasympathetic nervous system (PNS) index and the sympathetic nervous system (SNS) index

The PNS index was calculated using three parameters: RR, RMSSD, and SD1. The SNS index was calculated using the following three parameters: HR, SI, and the Poincaré Plot index SD2 expressed in normalized units.

### Psychological responses

Self-confidence, somatic anxiety, and cognitive anxiety were assessed using the CSAI-2R questionnaire. This inventory was selected for its three subscales and its widespread use in outdoor settings, ensuring a comprehensive and contextually relevant anxiety assessment. The CSAI-2R is a 17-item inventory, with each item scored on a Likert scale ranging from 1 (“not at all”) to 4 (“very much so”). Scores for each participant were combined to produce a score for each of the three subscales: (1) somatic anxiety (e.g., my heart is racing), (2) cognitive anxiety (e.g., I am concerned about performing poorly), and (3) self-confidence (e.g., I am confident because I can mentally picture myself reaching my goal). Just before the test during the first and second walks, the 17 statements were read and recorded by an examiner, and the participants verbally answered the appropriate number (1: Not at all, 2: Somewhat, 3: Moderately so, and 4: Very much so) to indicate how they felt at the moment (Cox et al., 2003).

### Statistical analysis

Descriptive statistics (mean ± standard deviation) were used to characterize physiological and psychological responses to low and high obstacles. Differences in the CSAI-2R scores between low and high obstacles were assessed between the first and second walks, while differences in HRV and respiratory markers were evaluated between the second and third walks, when walking speed was controlled. Repeated-measures ANOVA was conducted to assess statistical differences and effect sizes. Furthermore, Pearson correlation analysis was used to assess the relationship between physiological markers and the CSAI-2R results. Statistical significance was set at a *p*-value < 0.05. The correlations and squared association indices were interpreted as follows: strong effect: *R* ≥ 0.8; *R*^2^ ≥ 0.64, moderate effect: *R* ≥ 0.5; *R*^2^ ≥ 0.25, and low effect: *R* ≥ 0.2; *R*^2^ ≥ 0.04. All calculations were performed using Microsoft Excel and statistical software (IBM Corp., 2019, IBM SPSS Statistics for Windows, Version 28.0, Armonk, NY, USA).

## Results

### CSAI-2R

During the high-obstacle condition, the participants significantly (*p* < 0.01) decreased their self-confidence scores (↓3.0 ± 5.6) and increased both somatic anxiety (↑6.1 ± 5.7) and cognitive anxiety (↑2.9 ± 5.9) compared to the low-obstacle condition (Table 1).

**Table 1:** Mean ± SD responses for somatic anxiety, cognitive anxiety, and self-confidence from the CSAI-2R during walking on low- and high-obstacle courses at self-selected speeds.

CSAI-2R subscales	Low obstacle	High obstacle	*p*	*η*_p_^2^	*η*^2^G
Somatic anxiety	16.9 ± 4.8	23.0 ± 7.4	<0.001	0.543	0.292
Self-confidence	26.2 ± 7.1	23.2 ± 7.1	<0.001	0.220	0.072
Cognitive anxiety	16.6 ± 5.6	19.5 ± 8.2	<0.001	0.192	0.066

### Physiological response

#### Heart rate variability markers

Walking on the high-obstacle course significantly (*p* < 0.05) increased HR (↑32.6 ± 10.3 beats.min^−1^), SNS (↑7.2 ± 3.6), and SI (↑22.8 ± 15.2) and decreased NN50 (↓9.3 ± 17.1 beats), PNS (↓1.3 ± 0.5), RR (↓145.7 ± 55.8 ms), SDNN (↓13.5 ± 9.0 ms), RMSSD (↓9.9 ± 9.1 ms), SD1 (↓7 ± 6.4 ms), and DFA1 (↓0.1 ± 0.4) compared to the low-obstacle course (Table 2). The frequency-domain metrics did not show any significant differences (*p* > 0.05) between the low- and high-obstacle conditions: LFpow (n.u.) (↑3.5 ± 15.4), HFpow (n.u.) (↓3.6 ± 15.4), and LF/HF ratio (FFT) (↑1.1 ± 5.4) (Table 2). Based on the effect size magnitude, a strong effect (*η*_p_^2^ > 0.064) was found only for HR, RR, PNS index, SNS index, SI, and SDNN (Table 2).

**Table 2:** Mean ± SD responses for heart rate variability and respiratory markers during walking on low- and high-obstacle courses at the same walking speed.

Heart rate variability markers	Low obstacle	High obstacle	*p*	*η*_p_^2^	*η*^2^G
HR (beats.min^−1^)	102.7 ± 15.2	135.3 ± 18.0	<0.001	0.910	0.493
RR (ms)	597.1 ± 91.8	451.5 ± 61.7	<0.001	0.874	0.469
PNS index	−2.4 ± 0.8	−3.6 ± 0.6	<0.001	0.860	0.457
SNS index	4.7 ± 2.7	11.9 ± 5.4	<0.001	0.798	0.412
SI (Stress index)	22.4 ± 9.8	45.3 ± 20.7	<0.001	0.697	0.336
SDNN (ms)	23.4 ± 10.8	9.9 ± 6.9	<0.001	0.697	0.361
LFpow_FFT (ms^2^)	451.9 ± 370.9	91.8 ± 131.6	<0.001	0.536	0.299
RMSSD (ms)	16.1 ± 10.7	6.1 ± 4.2	<0.001	0.551	0.275
SD1 (ms)	11.4 ± 7.6	4.3 ± 3.0	<0.001	0.551	0.275
SD HR (bpm)	3.7 ± 1.0	2.6 ± 1.3	<0.001	0.397	0.186
HFpow_FFT (ms^2^)	147.2 ± 187.3	16.0 ± 26.4	<0.001	0.359	0.197
NN50 (beats)	10.1 ± 18.2	0.7 ± 1.9	0.001	0.231	0.117
DFA1	1.5 ± 0.2	1.4 ± 0.3	0.003	0.076	0.034
HFpow_FFT (n.u.)	20.7 ± 13.0	17.1 ± 10.9	0.410	0.052	0.022
LFpow_FFT (n.u.)	79.3 ± 13.0	82.8 ± 11.0	0.419	0.051	0.022
LF/HF ratio_FFT	6.0 ± 4.5	7.1 ± 4.9	0.549	0.038	0.013
Respiratory markers					
*V*_E_ (L.min^−1^)	22.5 ± 5.3	40.5 ± 8.5	<0.001	0.906	0.626
*V*O_2_ rel. (ml.kg^−1^.min^−1^)	9.2 ± 2.1	15.0 ± 3.2	<0.001	0.897	0.532
VT (L)	0.9 ± 0.2	1.3 ± 0.3	<0.001	0.893	0.326
*V*O_2_ (L.min^−1^)	0.7 ± 0.2	1.1 ± 0.3	<0.001	0.891	0.439
BF (breaths.min^−1^)	25.0 ± 4.0	33.4 ± 7.5	<0.001	0.643	0.332

The variables are sorted by the magnitude of *η*_p_^2^ in descending order.

HR, heart rate; RR, average time between consecutive heartbeats; PNS, parasympathetic nervous system index; SNS index, sympathetic nervous system index; SI, stress index; SDNN, mean of the standard deviations of RR intervals in 5-min segments; RMSSD, root mean square of successive differences between normal heartbeats; SD1, standard deviation 1; SD HR, standard deviation of heartbeats; NN50, parasympathetic activity; LFpow_FFT (ms^2^), low-frequency power measured by fast Fourier transform; HFpow_FFT (ms^2^), high-frequency power (0.15–0.4 Hz) measured using FFT; LFpow_FFT (n.u.), normalized value of low-frequency power expressed in normalized units; HFpow_FFT (n.u.), high-frequency power in normalized units; LF/HF Ratio (FFT), low-frequency power (LF) to high-frequency power (HF) ratio; DFA1, detrended fluctuation analysis; Alpha 1; V_E_, expiratory ventilation; VO_2_ rel., oxygen uptake; VO_2_, oxygen uptake; VT, tidal volume; BF, breath frequency.

#### Respiratory markers

Walking on the high-obstacle course significantly (*p* < 0.001) increased *VO_2_* (↑0.4 ± 0.1 L.min^−1^), V_E_ (↑ 18.1 ± 5.9 L.min^−1^), tidal volume (VT) (↑0.3 ± 0.1 L), and BF (↑8.5 ± 6.4 breaths.min^−1^) compared to walking on the low-obstacle course (Table 2). A strong effect of height (*η*_p_^2^ > 0.064) was found for all variables (Table 2).

### Relationships between physiological and psychological responses

No meaningful correlations were found between HRV, respiratory markers, and psychometric stress markers. Only HR and the SNS index showed a moderate positive correlation with *V*_E_ (*R* > 0.47) (Table 3).

**Table 3:** Correlations between heart rate variability (green), respiratory markers (red), and anxiety markers (blue).

Markers	PNS	SNS	SI	RR	SDNN	HR	VO_2_	VE	VT	SOMA	COGA	SC
PNS		−0.895^**^	−0.816^**^	0.985^**^	0.851^**^	−0.968^**^	−0.486^**^	−0.495^**^	−0.228	0.040	0.103	0.064
SNS	*−0.215*		0.983^**^	−0.892^**^	−0.823^**^	0.916^**^	0.469^**^	0.551^**^	0.218	−0.015	−0.056	−0.105
SI	*−0.127*	*0.970* ^**^		−0.803^**^	−0.809^**^	0.827^**^	0.431^**^	0.517^**^	0.212	−0.024	−0.058	−0.092
RR	*0.921* ^**^	*−0.118*	*−0.021*		0.819^**^	−0.988^**^	−0.485^**^	−0.505^**^	−0.216	0.034	0.095	0.067
SDNN	*0.816* ^**^	*−0.222*	*−0.233*	*0.674* ^**^		−0.785^**^	−0.365^**^	−0.323^*^	−0.188	0.159	0.180	−0.058
HR	*−0.694* ^**^	*0.674* ^**^	*0.504* ^**^	*−0.675* ^**^	*−0.443* ^**^		0.502^**^	0.549^**^	0.218	−0.019	−0.072	−0.091
VO2	*−0.097*	*0.392* ^**^	*0.319* ^*^	*−0.099*	*0.049*	*0.402* ^**^		0.821^**^	0.737^**^	−0.189	−0.358^**^	0.201
VE	*−0.147*	*0.470* ^**^	*0.392* ^**^	*−0.219*	*−0.013*	*0.495* ^**^	*0.620* ^**^		0.614^**^	0.093	−0.074	−0.078
VT	*−0.082*	*0.156*	*0.100*	*−0.138*	*0.100*	*0.241*	*0.404* ^**^	*0.355* ^**^		−0.366^**^	−0.454^**^	0.332^*^
SOMA	*0.270* ^*^	*0.023*	*0.052*	*0.323* ^*^	*0.313* ^*^	*−0.185*	*−0.153*	*−0.388* ^**^	*0.175*		0.784^**^	−0.805^**^
COGA	*0.124*	*0.128*	*0.176*	*0.239*	*0.084*	*−0.118*	*0.005*	*−0.348* ^**^	*0.178*	*0.733* ^**^		−0.776^**^
SC	*0.025*	*−0.196*	*−0.230*	*−0.053*	*0.007*	*−0.013*	*0.092*	*0.227*	*−0.016*	*−0.527* ^**^	*−0.596* ^**^	

The left diagonal matrix shows correlations between changes in physiological and psychological variables from low- to high-obstacle conditions. The right diagonal matrix shows correlations between these markers during the high-obstacle condition only. For clarity, only physiological markers of stress with a strong effect (*η*_p_^2^ > 0.64) were included in the correlation matrix.

** . Correlation is significant at the 0.01 level (2-tailed).

* . Correlation is significant at the 0.05 level (2-tailed).

PNS, parasympathetic nervous system index; SNS index, sympathetic nervous system index; SI, stress index; RR, average time between consecutive heartbeats (ms); SDNN, mean of the standard deviations of RR intervals in 5-min segments (ms); HR, heart rate (bpm); *V*_E_, expiratory ventilation (L.min^−1^); VO_2,_ oxygen uptake (L.min^−1^); VT, tidal volume (L); SOMA, somatic anxiety; COGA, cognitive anxiety; and SC, self-confidence. Distinct color shades indicate individual markers for improved clarity.

## Discussion

The results indicate that walking on a high-obstacle course significantly increased somatic and cognitive anxiety, sympathetic nervous activity, and metabolic demands, while reducing parasympathetic activity and self-confidence compared to walking on a low-obstacle course. Based on the magnitude of the effect size, the study identified key respiratory and HRV markers that determine the acute stress response to height. Surprisingly, it was found that physiological markers of stress and psychological metrics of somatic anxiety responses did not correlate, raising questions about their use and interpretation, for example, in outdoor adventure-based programs.

### CSAI-2R

The CSAI-2R is a commonly used and popular inventory for assessing anxiety in sport (Cox et al., 2003; Lundqvist and Hassmén, 2005; Martinent et al., 2010). In the current study, scores from all three dimensions of the questionnaire differed significantly between the low- and high-obstacle conditions, affirming that the CSAI-2R effectively reflects acute stress induced by height. Height induced nearly a threefold greater effect on somatic anxiety compared to cognitive anxiety or loss of self-confidence. However, somatic anxiety demonstrated only a low correlation with respiratory and HRV markers (*R* = 0.02–0.39), raising questions about the concurrent validity of this CSAI-2R dimension in assessing physiological changes related to the stress response. Similarly, Souza et al. (2019) did not find any meaningful correlation (*R* = 0.0–0.21) between somatic anxiety from the CSAI-2 and salivary cortisol or LF/HF ratio in the pre-competition state of sub-elite athletes. To the best of our knowledge, the psychometric properties of the CSAI-2R have primarily focused on reliability and factorial validity (Cox et al., 2003; Fernandes et al., 2013; Martinent et al., 2010), while the concurrent validity of the inventory dimensions, notably somatic anxiety, with physiological stress markers has not been established. In the current study, we observed the strongest correlation between VT and cognitive anxiety (*R* = −0.45) and between V_E_ and somatic anxiety (*R* = −0.39). However, these relationships were not consistent across both correlation matrices, and their significance is questionable.

The low correlations between somatic anxiety and physiological markers of acute stress responses suggest that self-reports using the CSAI-R do not reliably reflect objective physiological changes in the body. This finding supports the notion of a dissociation between cognitive-affective and autonomic elements, thereby contributing to debates about the extent to which subjective and physiological stress responses operate via partially independent pathways depending somewhat on individual differences, such as interoceptive accuracy (Thayer and Lane, 2000; Thayer and Lane, 2009; Critchley et al., 2004; LeDoux, 1996). Therefore, integrating psychometric tools with multi-dimensional physiological monitoring may provide a more accurate assessment of acute stress responses.

### HR and HRV

HR increased significantly during the high-obstacle task (103 ± 15 beats.min^−1^ for the low-obstacle task and 135 ± 18 beats.min^−1^ for the high-obstacle task, respectively). This increase was accompanied by a marked reduction in RR intervals (597.1 ± 91.8 ms to 451.5 ± 61.7 ms), reflecting heightened cardiac workload and a shift toward sympathetic dominance during the more stressful task, while intensity was held constant. These two HR markers showed the greatest differences between low- and high-obstacle conditions based on the *η*_p_^2^ magnitude.

Time-domain HRV measures showed significant reductions, with SDNN decreasing from 23.4 ± 10.8 ms to 9.9 ± 6.9 ms and RMSSD dropping from 16.1 ± 10.7 ms to 6.1 ± 4.2 ms, indicating diminished parasympathetic activity and reduced autonomic flexibility.

Frequency-domain HRV measures showed a slight decrease in parasympathetic activity and a minor increase in sympathetic modulation during the high-obstacle task. The LF/HF ratio also increased slightly, indicating a trend toward sympathetic dominance. However, the lack of significant changes suggests that sympathetic and parasympathetic shifts occurred proportionally, maintaining relative autonomic balance. These results emphasize that task difficulty has a stronger impact on time-domain HRV measures than on frequency-domain proportional metrics.

High-obstacle tasks led to a significant reduction in the PNS index (−2.4 ± 0.8 to −3.6 ± 0.6) and a nearly threefold increase in the SNS index (4.7 ± 2.7–11.9 ± 5.4). The stress index (SI) doubled during high-obstacle tasks (22.4 ± 9.8–45.3 ± 20.7). These results demonstrate a pronounced shift toward sympathetic dominance, consistent with the “fight-or-flight” response to the heightened physical and mental demands of the task.

### Respiratory responses

High-obstacle tasks significantly increased respiratory and metabolic demands, specifically with V_E_ rising from 22.5 ± 5.3 L.min^−1^ to 40.5 ± 8.5 L.min^−1^ from the low- to high-obstacle condition. This increase in V_E_ is much higher than the 7.7% increase reported during high versus low overground climbing (Gajdošík et al., 2020) in a similar population. It appears that the stress response to height is more pronounced when arm contact with a support is not possible and free space under the feet is present throughout the entire exercise. It should be noted that the increase in respiratory and metabolic responses was due to height exposure alone, as the speed of walking was kept the same as in the low-obstacle task. These responses also show that stress itself may increase the metabolic cost by 63%, as measured by VO_2_.

There are several limitations that must be acknowledged. The sample consisted of a relatively homogenous group of Czech university students, which limits the study’s generalizability to the broader population and to the general domain of education or therapeutic programs. Future research should include participants with greater heterogeneity to enable cross-cultural comparisons in the analyses presented.

Across all measures, there is a possibility for learning effects across multiple trials. Habituation, increased task familiarity, and task sequence may have combined to sequentially reduce physiological and psychological stress measures, most notably during the second low-obstacle trial. Furthermore, in the long term, it is unknown how longitudinal repeated exposures might influence stress and anxiety responses. Indeed, substantial variability in stress responses was observed across all variables, reflecting individual differences in stress adaptation and perception. This variability is likely influenced by factors such as prior experience with height (Ewert et al., 2016; Gajdošík et al., 2020; Giles et al., 2014), personality traits (Brouwer et al., 2015; Luo et al., 2023; Penley and Tomaka, 2002), social perceptions (Gillman et al., 2023), and other individual characteristics. In addition, external factors such as temperature, wind, light rain, humidity, and physiological needs (e.g., hunger, thirst, fatigue) may have also affected the outcomes (Ewert et al., 2016). Future research should explore the mediating or moderating roles of these confounding factors in stress responses and examine how adventure-based programs influence changes in stress markers. Although our study focused on HRV, respiratory measures, and self-reported anxiety to assess acute stress responses, future research could benefit from incorporating a broader set of physiological and micro-behavioral indicators. For example, facial micro-expression analysis (e.g., orbicularis oculi electromyography) and posture or gait dynamics may offer more nuanced real-time insights into participants’ experiences of height. Integrating these measures into multimodal models, along with temporal self-reporting, HRV, and ventilatory measures, would enable continuous, ecologically valid stress monitoring. This approach would allow for improved temporal resolution in detecting stress responses and could enhance intervention strategies in high-stress environments such as outdoor adventure programs.

## Conclusion

Walking at height induces a high level of perceived subjective stress, which is reflected in changes in both psychological and physiological markers. Using HR and respiratory markers such as *V*_E_, *V*O_2_, or VT, along with anxiety questionnaires during log walking, may be a relatively simple way to assess stress coping in outdoor adventure programs. This approach may also serve as a simple method for evaluating the effectiveness of these programs. However, combining physiological and psychometric tools is essential, as they measure different dimensions of the stress response.

## Data Availability

The original contributions presented in the study are included in the article/supplementary material, further inquiries can be directed to the corresponding author.
